# Echocardiography‐Assisted Stenting of the Ductus Venosus in Infracardiac TAPVC: Report of a New Technique and Review of the Literature

**DOI:** 10.1002/ccd.70631

**Published:** 2026-04-23

**Authors:** Simon Schmid, Michael Hofbeck, Johannes Nordmeyer, Ralf Rauch, Jörg Michel

**Affiliations:** ^1^ Department of Pediatric Cardiology, Pulmonology and Intensive Care University Children's Hospital Tübingen Germany; ^2^ Pediatric Department of Rems‐Murr Hospital Winnenden Germany

**Keywords:** congenital heart disease, ductus venosus, extremely low birth‐weight infant, stenting, TAPVC

## Abstract

Neonates with infracardiac total anomalous pulmonary venous connection (TAPVC) frequently require urgent treatment. If surgical repair is contraindicated due to extreme prematurity, interventional stenting of the ductus venosus (DV) has been introduced successfully to postpone surgery. We report a new technique of transjugular stenting in an 890‐g premature infant who presented with infracardiac TAPVC and functional closure of the DV. The DV was successfully crossed with a wire and stented under echocardiographic control. The case underlines the importance of echocardiography in diagnosing and managing DV obstruction. Different interventional strategies for DV‐stenting in these complex patients are briefly reviewed.

## 1 Introduction

In most patients with infracardiac total anomalous pulmonary venous connection (TAPVC) pulmonary venous flow drains via a vertical vein (VV) into the portal vein (PV) system [1]. Patency of the ductus venosus (DV) is essential to bypass hepatic circulation and to maintain unobstructed pulmonary venous return. Closure of the DV results in life‐threatening pulmonary venous obstruction with subsequent pulmonary edema and hypoxemia [1]. Immediate surgical correction with anastomosis of the pulmonary veins to the left atrium may be unfeasible in extremely premature or critically ill neonates [2]. Catheter‐based interventions to abolish obstruction caused by a stenotic DV have emerged as therapeutic alternatives in selected cases. We report a previously undescribed technique for emergency treatment of a functionally closed DV in an extremely premature infant with infracardiac TAPVC to the PV and discuss therapeutic strategies for the management of this complex malformation (Table 1).

**Table 1:** Overview of published case reports on DV stenting in TAPVC patients.

Author	Birth weight	Age of inter‐ven‐tion	Access	Stent type	Stent	Stent dimension [mm × mm]	VV stenting	Antico‐agulation	Re‐stenosis intervall	Age of TAPVC repair	Weight at TAPVC repair	Diagnosis	DV closure
Meadows et al. [5]	3900 g	3 d	Fem	n.a.	n.a.	n.a.	Yes	n.a.	n.a.	8 d	3900 g	Infracardiac TAPVC	Spontaneous
Higaki et al. [6]	3000 g	3 d	Umb	Cor	GFX Micro (BMS, Guidant/Abbott)	3 × 24 + 3 × 18 + 3 × 18	No	Aspirin	1 m	4 m	n.a.	UVH, PA, infracardiac TAPVC	n.a.
Higaki et al. [6]	2580 g	1 d	n.a.	Per	Palmaz (BMS, Cordis)	8 × 24	No	n.a.	No	3 m	n.a.	UVH, PA, infracardiac TAPVC	n.a.
Oonishi et al. [7]	1500 g	1 d	n.a.	Cor	n.a. (BMS)	3.5 × 13 + 3.5 × 13	No	n.a.	35 d	53 d	n.a.	Heterotaxy, PA, Infracardiac TAPVC	Spontaneous
Rothman et al. [8]	1700 g	2 d	Umb	Cor	n.a.	4 × 20	No	Heparin	14 d	3 w	n.a.	Mixed TAPVC	Spontaneous
Rothman et al. [8]	1500 g	13 d	Umb	Cor	Duet (BMS, Guidant/Abbott)	3.5 × 13	No	Heparin	No	5 w	n.a.	Infracardiac TAPVC	Spontaneous
Rothman et al. [8]	3200 g	2 d	Umb	Cor	Liberte (BMS, Boston Scientific)	4 × 24	No	Heparin	No	4 d	n.a.	Infracardiac TAPVC	AVP I (Abbott)
Burkhardt et al. [9]	1270 g	16 d	Jug	Cor	Driver (BMS, Medtronic)	4.5 × 9	No	Aspirin	2 m	3 m	2000 g	Infracardiac TAPVC	n.a.
Hope et al. [10]	940 g	6 d	Umb + jug	Cor	Resolute Onyx (DES, Medtronic)	5 × 12	Yes (initialy)	n.a.	no	3 m	2700 g	DORV, infracardiac TAPVC	Spontaneous
Chamberlain et al. [11]	1900 g	10 d	Jug	Cor	n.a. (BMS)	4 × 18 + 4×12	No	Enoxaparin	30 d	60 d	2800 g	Infracardiac TAPVC	n.a.
Kisamori et al. [12]	n.a.	n.a.	n.a.	Cor	BioMatrix (DES, Biosensors)	3.5 ×24	No	n.a.	n.a.	n.a.	n.a.	UVH, PS, infracardiac TAPVC	n.a.
Kisamori et al. [12]	n.a.	n.a.	n.a.	Cor	Resolute Onyx (DES, Medtronic)	4 × 22	No	n.a.	n.a.	n.a.	n.a.	UVH, PA, infracardiac TAPVC	n.a.
Kisamori et al. [12]	n.a.	n.a.	n.a.	Cor	Resolute Integrity (DES, Medtronic)	3.5 × 30	No	n.a.	n.a.	n.a.	n.a.	UVH, PS, infracardiac TAPVC	n.a.
Kisamori et al. [12]	n.a.	n.a.	n.a.	Cor	Nobori (DES, Terumo)	3.5 × 18	No	n.a.	n.a.	n.a.	n.a.	UVH, mixed TAPVC	n.a.
Imai et al. [13]	2296 g	1 d	Jug	Cor	BMX‐J (DES, Biosensors)	3.5 × 24	Yes (second)	Warfarine + aspirin	n.a.	77 d	3100 g	Heterotaxy, UVH, PA, Infracardiac TAPVC	Flipper PDA coil 5 mm (Cook Medical), Azur coil 4mm × 5cm (Terumo)
Imai et al. [13]	1998 g	11 d	Jug	Cor	Resolute Integrity RX (DES, Medtronic)	3.5 × 30	No	Warfarine + aspirin	77 d	113 d	2800 g	Heterotaxy, UVH, PA, infracardiac TAPVC	Azur coil 4mm × 5cm (Terumo)
Imai et al. [13]	3681 g	1 d	Jug	Cor	Resolute Integrity RX (DES, Medtronic)	4 × 22	No	Warfarine + aspirin	n.a.	14 d	3700 g	Heterotaxy, UVH, PS, mixed TAPVC	Flipper PDA coil 5 mm (Cook Medical), Azur CX coil 6mm × 20cm (Terumo), AVP II 4–6 mm (Abbott)
Said et al. [14]	3000 g	1 d	Umb	Cor	Resolute Onyx (DES, Medtronic)	5 × 15	Yes (initialy)	n.a.	No	n.a.	3000 g	infracardiac TAPVC	Flipper AVP II 8 mm (Abbott)
						5 × 12							
Cameron et al. [15]	935 g	17 d	Jug	Cor	Resolute Onyx (DES, Medtronic)	4 × 12 + 4 × 16	No	aspirin	6 w	2 m	2000 g	infracardiac TAPVC	APO 4 × 4mm (Abbott)
					Formula 418 (BMS, Cook Medical)								
George et al. [16]	1775 g	21 d	Hep	Per	Formula 418 (BMS, Cook Medical)	4 × 16	No	n.a.	2w + 4w	No	—	infracardiac TAPVC	n.a.
Ito et al. [17]	902 g	1 d	Umb	No	No	No	No	n.a.	No	21 d	n.a.	CAVV, DORV, Infracardiac TAPVC	no
Mejia et al. [18]	1700 g	14 d	Umb	Cor	n.a.	n.a.	No	n.a.	4 m	6 m	n.a.	mixed TAPVC	n.a.
Mejia et al. [18]	1900 g	17 d	Jug	Cor	n.a.	n.a.	Yes (second)	n.a.	2 d	2 m	n.a.	Infracardiac TAPVC	n.a.
Current	890 g	15 d	Jug	Cor	Pro‐Kinetic Energy (BMS, Biotronik)	3.5 × 13	No	Enoxaparin	5 d + 7 d	13 w	2100 g	Infracardiac TAPVC	AVP II 6 mm (Abbott)

Abbreviations: APO, Amplatzer Piccolo Occluder; AVP, Amplatzer Vascular Plug; BMS, bare‐metal stent; CAVV, common atrioventricular valve; cor, coronary stent; d, days; DES, drug‐eluting stent; DORV, double outlet right ventricle; DV, ductus venosus; Ex, death; fem, femoral access; hep, transhepatic access; jug, jugular access; n.a., not applicable; PA, pulmonary atresia; per, peripheral stent; PS, pulmonary stenosis; m, months; umb, umbilical access; UVH, univentricular heart; VV, vertical vein; w, weeks.

## 2 Case

**Figure 1:**
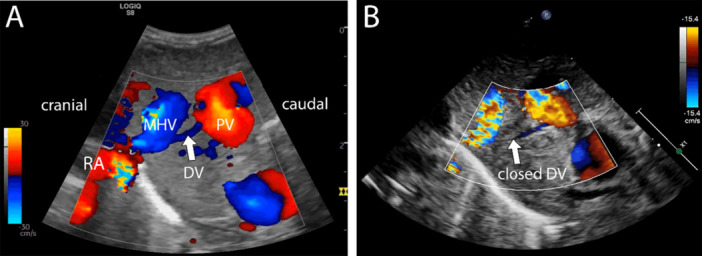
Color Doppler echocardiography (longitudinal view of the upper abdomen): (A) Patent DV showing severe constriction at the referring hospital (MHV = middle hepatic vein, PV = portal vein, RA = right atrium); (B) closed DV after transfer. [Color figure can be viewed at wileyonlinelibrary.com]

**Figure 2:**
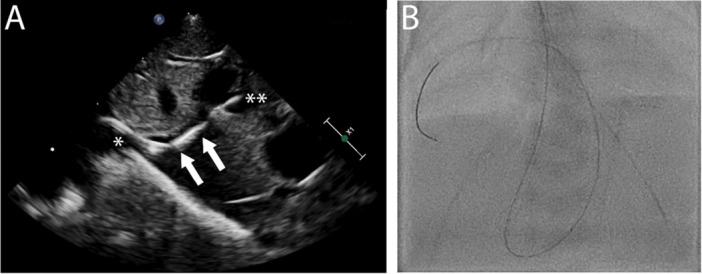
(A) Echocardiographically guided advancement (longitudinal view of the upper abdomen) of the wire from the inferior vena cava (IVC, *) toward the portal vein (PV, **), across the closed ductus venosus (DV, two arrows). (B) Fluoroscopic image demonstrating the final wire position following passage of the DV and PV via the descending vertical vein to the right inferior pulmonary vein. [Color figure can be viewed at wileyonlinelibrary.com]

**Figure 3:**
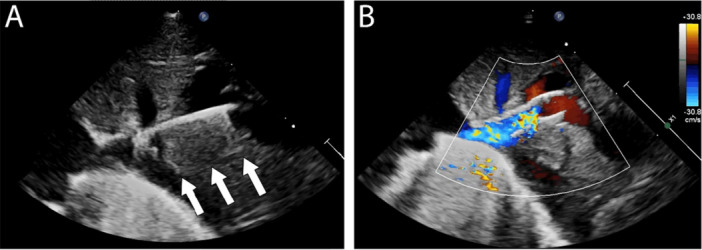
(A) Placement of the stent within the ductus venosus (longitudinal view of the upper abdomen). Prior to inflation, the position of the stent can be determined by its posterior echo‐extinction (arrows). (B) Color Doppler echocardiography after balloon inflation shows restored flow across the stented DV. [Color figure can be viewed at wileyonlinelibrary.com]

**Figure 4:**
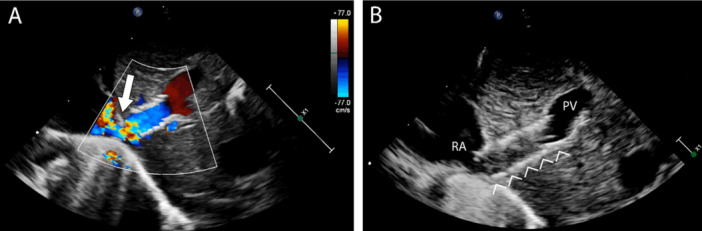
(A) Significant shortening of the stent (color Doppler echocardiography, longitudinal view of the upper abdomen) following redilatation. Protrusion of hepatic tissue resulted in stenosis of the stent at its proximal end (arrow). (B) The obstruction was managed by implantation of a second overlapping stent (arrowheads). PV = portal vein, RA = right atrium. [Color figure can be viewed at wileyonlinelibrary.com]

The male infant was born at 28 weeks of gestation (birth weight: 890 g). Echocardiography at the referring hospital revealed infracardiac TAPVC connecting to the PV. In the presence of a large non‐obstructive DV, the neonate was initially stable. On Day 15 of life, the infant developed rapidly progressive obstruction of the DV, resulting in significant deterioration of his respiratory situation requiring intubation and mechanical ventilation (Figure 1A). Following referral to our hospital, targeted Doppler‐echocardiography in a subcostal longitudinal view revealed complete closure of the DV (Figure 1B). No detectable flow signal was observed between the portal venous system and the inferior vena cava (IVC). Given the substantial pressure gradient between the congested portal venous system and the low‐pressure systemic venous circulation, even a severely stenotic but still patent DV would be expected to show a detectable Doppler signal. Venous return to the systemic circulation was therefore most likely maintained via intrahepatic drainage through the hepatic sinusoids into the hepatic veins and subsequently into the IVC. The resulting severe pulmonary venous congestion led to pronounced pulmonary edema, with transcutaneous oxygen saturation of approximately 70% at the time of intervention, despite mechanical ventilation with 100% oxygen. After a multidisciplinary discussion, there was consensus that surgery would be associated with an unacceptable risk, and the decision was made to proceed with an interventional approach. Informed consent was obtained from both parents. Under general anesthesia in the cardiac catheterization laboratory (procedure weight: 900 g), percutaneous access was established via the right internal jugular vein using a 4 Fr sheath. Because no Doppler‐detectable flow was present within the DV and given the emergent clinical situation in an extremely premature infant, contrast angiography was intentionally not performed in order to minimize contrast exposure and prioritize the intervention. Therefore, the fibrous strand of the closed DV, the IVC, and the PV were visualized. A 2.7 Fr microcatheter (Progreat, Terumo) was advanced through a 4 Fr guiding catheter (GLIDECATH Angle, Terumo) into the IVC. Under echocardiographic guidance, the wire of the microcatheter was directed toward the closed DV (Figure 2A). Fluoroscopy was used selectively to confirm microcatheter position within right inferior pulmonary vein over the descending vertical vein (Figure 2B). The 0.021” wire was exchanged for a 0.014” peripheral guidewire (Glidewire Advantage, Terumo). This guidewire was used to advance a bare metal coronary stent (PRO‐Kinetic Energy 3.5 × 13 mm, Biotronik) into the DV (Figure 3A). Stent length had been chosen according to the sonographic measurement, and stent diameter was selected based on the sonographic estimate of the target vessel caliber and the infant's extremely low body size, in line with diameters reported in comparable cases. Stent positioning and expansion were monitored exclusively by echocardiography, confirming restored and unobstructed DV flow by color Doppler (Figure 3B). During the initial intervention and post‐interventionally, no contrast angiography was performed; fluoroscopy was used solely for stent visualization and documentation. Prophylactic anticoagulation was initiated with subcutaneous enoxaparin (1.5 mg/kg/day). Three weeks later, in‐stent restenosis occurred and was treated by balloon dilation with a 6 × 20 mm balloon. Proximal obstruction caused by hepatic tissue (Figure 4A) required implantation of a second bare metal stent (Express LD, Boston Scientific, 6 × 15 mm) (Figure 4B). During the repeat catheterization, angiography was performed to assess in‐stent restenosis and the procedural result.

At the age of 13 weeks (weight 2.1 kg), surgical repair with anastomosis of the pulmonary vein confluence to the left atrium was performed successfully. Given persistent portosystemic shunting and a mildly elevated serum ammonia level (73 µmol/L), DV was closed interventionally 2 weeks later using a 6 mm Amplatzer Vascular Plug II (Abbott), after pulmonary hypertension had been excluded by invasive measurement demonstrating a mean pulmonary artery pressure of 15 mmHg. At 6 months, repeat cardiac catheterization revealed a pressure gradient of 3 mmHg in the IVC at the previous DV site, which resolved after balloon dilatation. Pulmonary artery pressures were normal, and there was no evidence of pulmonary venous obstruction.

## 3 Discussion

TAPVC represents a rare but critical congenital malformation characterized by abnormal drainage of the pulmonary veins into the right atrium or systemic venous circulation. The clinical condition of patients with infracardiac TAPVC and drainage of the descending vein into the PV system is closely linked to unobstructed patency of the DV. The DV is a structure of the fetal circulation that allows unobstructed flow of the umbilical vein to the IVC and the right atrium. While the DV usually closes during the first few days of life in healthy term neonates, its closure may be delayed in the presence of prematurity, persistent pulmonary hypertension, or hypoxia [3, 4]. In premature infants with infracardiac TAPVC, therapy can be delayed as long as the DV remains nonobstructive [4]. In the absence of a patent DV, portal venous blood may still reach the systemic circulation through the hepatic microcirculation via hepatic sinusoids and hepatic veins, although this pathway is less efficient, leads to severe pulmonary venous congestion and may contribute to rapid clinical deterioration in patients with infracardiac TAPVC [3]. However, our case shows that constriction and closure of the DV may develop very rapidly within 24 h. Therefore, this conservative approach requires very close monitoring of the DV by repeated echocardiography.

Definitive repair of infracardiac TAPVC requires cardiopulmonary bypass and is generally well tolerated in term‐born neonates, whereas extreme prematurity, low birth weight, or critical condition substantially increase operative risk. In such situations, interventional treatment has emerged as an effective bridge to surgery, even in extremely low birth‐weight infants, to allow maturation toward term‐equivalent age. Successful stenting of the DV has been described so far in 23 cases [5, 6, 7, 8, 9, 10, 11, 12, 13, 14, 15, 16, 17, 18]. In one additional case, the procedure was aborted due to a bleeding complication [17]. The right internal jugular and umbilical veins were the most common routes for percutaneous access (8 of 23 cases each), while a combined approach was reported once and ultimately required jugular venous access due to the underlying anatomy [10]. The jugular route provides a direct path to the IVC and a very favorable angle to enter the DV [9, 10, 11, 13, 15, 18], whereas umbilical access is mainly feasible in the early postnatal period when an umbilical venous catheter (UVC) has been positioned across the DV [6, 7, 10, 14, 17, 18]. In similar anatomic configurations, maintaining a UVC positioned across the DV may be lifesaving and can facilitate catheter‐based palliation; however, this option is limited to the early postnatal period and requires appropriate catheter positioning. A transhepatic approach, though possible in neonates [16], appears to be associated with a high procedural risk in premature low birth‐weight infants.

Accurate preprocedural imaging of the anatomy is essential for safe and effective intervention. This includes a detailed assessment of DV morphology, its connection to the portal venous system, and potential stenoses in the descending vertical vein. Most previous reports relied on an angiographic definition of the anatomy. However, most of the required information may be gained by meticulous two‐dimensional and Doppler‐echocardiographic evaluation [19, 20, 21], supported by bibliometric data confirming the central diagnostic role of echocardiography in TAPVC [22]. Since echocardiography alone may be insufficient to fully delineate pulmonary venous anatomy in up to one‐third of cases, especially in complex variants, structured imaging protocols are required to ensure diagnostic accuracy [23]. Advanced imaging based on computed tomographic angiography may provide valuable 3‐dimensional information, but will be restricted to cases with enough time to perform this procedure [18]. In our case, following the complete closure of the DV, we had to rely completely on echocardiographic and sonographic imaging. The fibrous strand of the DV, which had closed within the preceding 12 h, was clearly visualized between the IVC and the PV, allowing exact measurement of its length. Both probing of the DV and subsequent stent placement and inflation were performed under sonographic visualization.

According to our review of the literature, bare‐metal and drug‐eluting stents were used with equal frequency (8 of 23 cases each, 34.8%, including one patient receiving both), with a mean diameter of 4.1 mm (range 3–8 mm) and a mean length of 19.0 mm (range 9–30 mm) [5, 6, 7, 8, 9, 10, 11, 12, 13, 14, 15, 16, 17, 18]. Experience with drug‐eluting stents and bare‐metal stents remains limited [10, 12, 13, 14, 15], and no drug‐eluting stent‐specific adverse effects have been reported in the published neonatal cases to date. After successful DV recanalization, all patients required close echocardiographic surveillance to detect possible restenosis of the DV, which was reported in 56.5% of the cases, occurring at a mean interval of 6.8 weeks (range 2−18 weeks) after the initial intervention [6, 7, 8, 9, 11, 13, 15, 16, 18]. As in our case, restenosis can be managed by balloon dilatation or implantation of a second stent.

Different strategies for prophylactic antithrombotic treatment following DV‐stenting have been reported, reflecting variations in clinical practice and limited experience in this rare setting. Reported approaches include aspirin monotherapy, dual therapy with phenprocoumon and aspirin, and low‐molecular‐weight heparin (LMWH) [11]. We opted for treatment with the LMWH enoxaparin because of its predictable pharmacokinetics. Under this regimen, the DV remained patent even beyond corrective surgery, resulting in postoperative portosystemic shunting. In accordance with most previous reported cases, elective interventional closure of the DV was performed to avoid side effects of portosystemic shunting, including hyperammonemia and liver dysfunction [13].

## 4 Conclusion

DV stenting is a viable bridge to surgery in extremely low birth‐weight infants with infracardiac TAPVC. In the presence of a recently closed DV, sonographic imaging can guide wire crossing and successful reopening by placement of a stent.

## Funding

The authors have nothing to report.

## Ethics Statement

Written informed consent was obtained from the parents of the patient for publication of the details of their medical case and any accompanying images. This study protocol was reviewed and approved by the ethics committee at the Medical Faculty of Eberhard Karls University and at the University Hospital Tübingen, approval number 838/2023A.

## Conflicts of Interest

The authors declare no conflicts of interest.

## Data Availability

The data that support the findings of this study are available on request from the corresponding author. The data are not publicly available due to privacy or ethical restrictions.
